# Evaluating the antibody response elicited by diverse HIV envelope immunogens in the African green monkey (Vervet) model

**DOI:** 10.1038/s41598-024-63703-7

**Published:** 2024-06-10

**Authors:** Thandeka Moyo-Gwete, Frances Ayres, Nonkululeko B. Mzindle, Zanele Makhado, Nelia P. Manamela, Simone I. Richardson, Dale Kitchin, Strauss van Graan, Joritha van Heerden, Nishal Parbhoo, Gerald K. Chege, Penny L. Moore

**Affiliations:** 1 SA MRC Antibody Immunity Research Unit, School of Pathology, Faculty of Health Sciences, University of the Witwatersrand, Johannesburg, South Africa; 2 Centre for HIV and STIs, National Institute or Communicable Diseases (NICD) of the National Health Laboratory Service (NHLS), Johannesburg, South Africa; 3 Primate Unit and Delft Animal Centre, Centre and Platform Office, South African Medical Research Council, Cape Town, South Africa; 4 Department of Life and Consumer Sciences, College of Agriculture and Environmental Sciences, University of South Africa, Johannesburg, South Africa; 5 Division of Medical Virology, Department of Pathology, University of Cape Town, Cape Town, South Africa; 6 Centre for the AIDS Programme of Research in South Africa (CAPRISA), Durban, South Africa

**Keywords:** Adaptive immunity, Experimental models of disease

## Abstract

African Green (Vervet) monkeys have been extensively studied to understand the pathogenesis of infectious diseases. Using vervet monkeys as pre-clinical models may be an attractive option for low-resourced areas as they are found abundantly and their maintenance is more cost-effective than bigger primates such as rhesus macaques. We assessed the feasibility of using vervet monkeys as animal models to examine the immunogenicity of HIV envelope trimer immunogens in pre-clinical testing. Three groups of vervet monkeys were subcutaneously immunized with either the BG505 SOSIP.664 trimer, a novel subtype C SOSIP.664 trimer, CAP255, or a combination of BG505, CAP255 and CAP256.SU SOSIP.664 trimers. All groups of vervet monkeys developed robust binding antibodies by the second immunization with the peak antibody response occurring after the third immunization. Similar to binding, antibody dependent cellular phagocytosis was also observed in all the monkeys. While all animals developed potent, heterologous Tier 1 neutralizing antibody responses, autologous neutralization was limited with only half of the animals in each group developing responses to their vaccine-matched pseudovirus. These data suggest that the vervet monkey model may yield distinct antibody responses compared to other models. Further study is required to further determine the utility of this model in HIV immunization studies.

## Introduction

More than 40 years after the initial discovery of the human immunodeficiency virus (HIV), there is still no effective vaccine to prevent infection. There is, therefore, still a need for novel immunogens which can trigger the development of broadly neutralizing antibodies (bNAbs) which are able to prevent entry of the diverse HIV subtypes^1^. The HIV Envelope (Env) is the sole target of bNAbs, making it an attractive vaccine target^2^ and it has been modified in various ways to increase stability and solubility including the SOSIP.664 trimer format^3^.

The trimer derived from the BG505 HIV strain has been the gold-standard SOSIP.664 trimer due to its intrinsic ability to form stable trimers in a preferentially closed conformation that are recognized by bNAbs^3,4^. Despite this, animal studies using BG505 and other SOSIP.664 trimers have not consistently yielded potent cross-neutralizing responses towards neutralization-resistant (Tier 2) HIV strains in rabbit, guinea pig and non-human primate (NHP) models^5–8^. One of the limitations of SOSIP trimers as immunogens is that the base of the trimer, which is an epitope for non-neutralizing responses, is highly immunogenic and skews the immune response towards undesirable, non-neutralizing, binding responses^9–11^.

Macaques offer an attractive model for HIV immunogen pre-clinical testing as they are genetically similar to humans^12^. Whilst the rhesus macaque is globally well-accepted as the NHP model of choice for HIV vaccine research, the African green (vervet) monkey may represent a valuable and alternative model which is underutilized for immunogenicity studies. Vervet monkeys (*Chlorocebus aethiops*) have been essential models for HIV pathogenesis studies as these NHPs, unlike macaques, are able to naturally control simian immunodeficiency virus (SIV) and do not progress to acquired immune deficiency syndrome (AIDS)^13^. Macaques are large animals requiring specialized containment facilities, are not easily accessible and their acquisition and upkeep are expensive as compared to vervet monkeys. This makes the use of macaques prohibitive for resource limited settings which would benefit from a cheaper, more readily available NHP model, providing an alternative measure of immunogenicity for potential HIV vaccines. Moreover, vervet monkeys breed throughout the year, unlike the rhesus monkeys which are seasonal breeders, making them more attractive for research-purpose breeding. Vervet monkeys have small body sizes and can be housed in social groups, making their maintenance in research facilities cheaper and more manageable than larger NHPs^14^.

Thus, we explored the use of vervet monkeys as a NHP immunogenicity model to evaluate the elicitation of binding and neutralizing antibodies as well as Fc effector functions in response to HIV Env trimer immunogens. As Env proteins from individuals who develop bNAbs have been shown to have the ability to “imprint” bNAb responses^15^, we designed a subtype C SOSIP.664 trimer derived from a virus isolated 8 weeks post-infection from a person living with HIV (PLWH) who developed a broadly neutralizing response targeting the V3/glycan-site^16^. We tested the ability of this trimer to induce anti-HIV binding, neutralizing and Fc-effector antibodies and compared this to BG505 immunization, as the current “gold standard”. In addition, we tested the ability of a cocktail of trimers (subtypes C/C/A) to induce antibody responses upon immunization to determine whether the use of heterogeneous Env trimers could elicit superior responses compared to a single immunogen. We tested a combination of CAP255, BG505 and a CAP256 trimer from an individual who developed potent bNAb responses towards the V2/apex^17,18^. After four immunogen doses, all the vervet monkeys elicited robust binding antibodies and phagocytic activity. However, neutralizing antibody responses were only elicited towards neutralization-sensitive (Tier 1) viruses and limited autologous or Tier 2 neutralization was observed, even after BG505 immunization. This is unlike BG505 immunization in macaque models^19^. Altogether, our data suggest that although the vervet monkey model is invaluable as an HIV pathogenesis model, in this study, only weak and infrequent neutralizing antibody responses were detected. Further immunization studies using vervet monkeys should be explored using varying parameters to determine the utility of the model.

## Results

### Design and characterization of the CAP255 SOSIP.664 trimer for immunization studies

**Figure 1:**
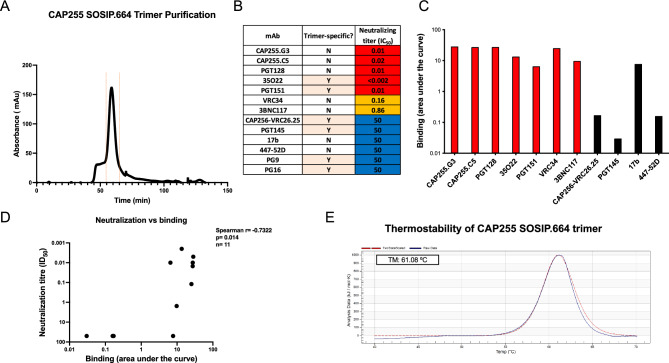
Design, expression and characterization of the CAP255 trimer. (**A**) The The CAP255 SOSIP.664 trimer was expressed in HEK 293F cells and purified by nickel affinity chromatography followed by size exclusion chromatography. The purity of the trimer was assessed by SDS-PAGE. (**B**) An HIV pseudovirus-based neutralization assay was used to determine the neutralization titer of monoclonal antibodies against the CAP255 pseudovirus. IC_50_ values are shown in μg/ml with resistance being > 50 μg/ml (blue) and increasing sensitivity from 0.1–0.9 μg/ml (orange) to < 0.09 μg/ml (red). Trimer-specific antibodies are labelled as Y (yes) and monomer-specific antibodies are labelled as N (No). (**C**) Monoclonal antibody binding responses towards the trimer were assessed by ELISA. An antibody bead depletion assay was used to assess the binding of conformational antibody, PGT151 (**D**) A Spearman correlation was conducted in Graphpad Prism v9.5.1 to assess the relationship between binding and neutralizing titers. (**E**) Differential scanning calorimetry was used to assess the melting temperature of the CAP255 trimer.

We successfully designed, expressed and purified a subtype C trimer from a PLWH, CAP255, from the CAPRISA 002 acute infection cohort. CAP255 developed a broadly neutralizing response 1 year post-infection, which targeted the V3/glycan-site^16^. We designed a CAP255 SOSIP.664 trimer based on a well characterized early 8 week *env* sequence and introduced the standard SOSIP.664 mutations^3^. The CAP255 SOSIP.664 trimer expressed well, with very little aggregation or dimer/monomer formation (Fig. 1A, Supp. Fig. S1). We compared the neutralization profile of the CAP255 pseudovirus (Fig. 1B) with the binding profiles of the same monoclonal antibodies to the corresponding trimeric protein (Fig. 1C) and found that there was a strong correlation between binding and neutralization (Fig. 1D). These data confirm that the CAP255 SOSIP.664 is a robust antigenic mimic of the corresponding pseudovirus and is conformationally intact. The trimer was bound by conformational antibodies including 35,022 and bead depletion experiments showed that PGT151 bound strongly to the trimer (Fig. 1C). These data confirmed that the trimer was in the optimal, closed conformation. Lastly, we performed differential scanning calorimetry to determine the thermostability of the trimer. The CAP255 SOSIP.664 trimer had a high melting temperature of 61 °C (Fig. 1E). The CAP255 trimer melting temperature was lower than the other trimers with the well-characterized BG505 SOSIP.664 trimer having a reported melting temperature in the range of 67–68 °C^3,20,21^ and the CAP256.wk34.c80 trimer with a reported melting temperature of 77 °C^22^; however, at 61 °C it can still be regarded as thermostable.

### Vervet monkey HIV Env trimer immunizations elicit robust binding antibody responses

**Figure 2:**
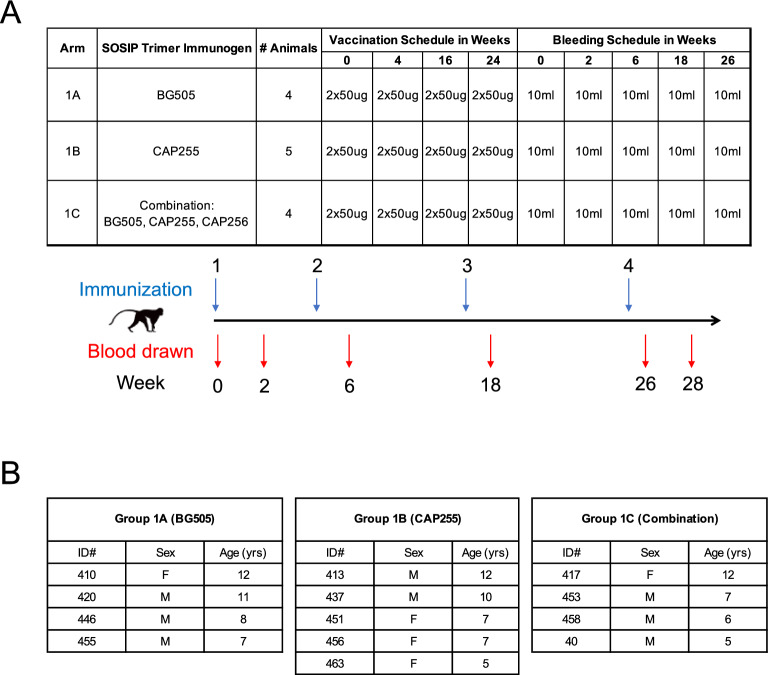
Immunization and sampling schedule for the vervet monkey model. (**A**) Vervet monkeys were assigned into groups with Group 1A receiving BG505 SOSIP.664 only, Group 1B receiving CAP255 SOSIP.664 only and Group 1C receiving a combination between BG505, CAP255 and CAP256.SU SOSIP.664 trimers. Each group received their immunogen(s) through 2 subcutaneous injections of 50 μg (total = 100 μg) of trimer during each vaccination and 10 ml of blood was collected at each blood draw. (**B**) Group 1A (n = 4), Group 1B (n = 5) and Group 1C (n = 4) each had a distribution of male and female vervet monkeys within an age range of 5–12 years of age.

**Figure 3:**
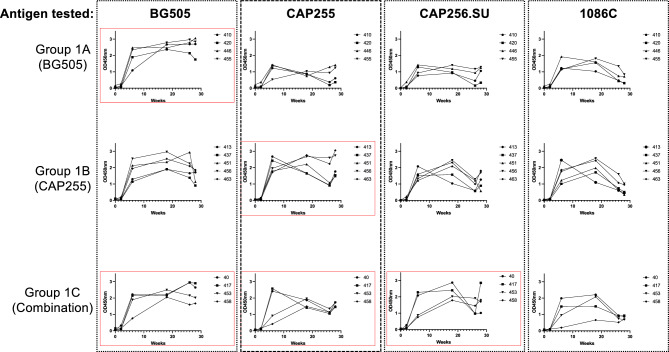
Immunization with SOSIP.664 trimers elicits cross-reactive binding antibodies. The presence of HIV Env-specific binding antibodies in plasma from BG505 (Group 1A), CAP255 (Group 1B) and or BG505, CAP255 and CAP456.SU combination (Group 1C) immunized vervet monkeys against homologous (red boxes) and heterologous antigens was assessed using an in-house ELISA. Binding is represented by an OD450nm value.

We embarked on an immunogenicity study to investigate whether HIV-specific antibodies could be elicited in vervet monkeys upon immunization with either BG505 (Group 1A), CAP255 (Group 1B) or a cocktail of BG505, CAP255 and CAP256.SU (based on the CAP256.wk34.c80 virus^23^) (Group 1C) SOSIP.664 trimers. A total of 13 vervet monkeys were assigned to the 3 immunization groups (n = 4 for each group except the CAP255 group with n = 5) (Fig. 2B). All animals were between the ages of 5–12 years and each group had female and male monkeys (Fig. 2B). All animals were confirmed to be SIV-naive. Each animal received four immunizations over a period of 24 weeks with the assigned test immunogen(s) (Fig. 2A). A total of six blood samples were collected from each animal with blood draws at baseline, two weeks after each immunization and a final collection 4 weeks after the fourth immunization (Fig. 2A). We tested the vervet monkey plasma at baseline and 2 weeks after each immunization for binding antibodies. We tested binding towards the Env trimers that the NHPs were immunized with (BG505, CAP255 and CAP256.SU SOSIP.664 trimers) as well as the heterologous subtype C HIV SOSIP.664 Env, 1086C, to determine the level of cross-reactive HIV-specific binding antibodies elicited upon vaccination (Fig. 3).

**Figure 4:**
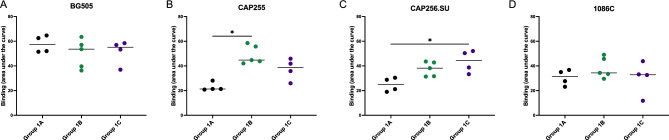
Comparison of binding antibody responses between immunization groups. Binding antibody responses measured through ELISA for each timepoint tested. The area under the curve was calculated for each animal across all timepoints and this was compared between Groups 1A, 1B and 1C for each antigen tested: (**A**) BG505, (**B**) CAP255, (**C**) CAP256.SU and (**D**) 1086C. Statistical significance was calculated in Graphpad Prism v9.5.1 using the Kruskal–Wallis test with Dunn’s multiple comparison test where *p < 0.05, **< 0.01, ***< 0.001, ****< 0.0001.

All animals had no detectable anti-HIV binding antibodies at baseline as expected. A high magnitude of binding antibodies were already detectable after the second immunization with OD450nm values of up to 2.580, generally peaking after the third immunization with OD450nm values up to just under 3. These responses persisted up to one month after the fourth immunization (Fig. 3). We compared antibody responses over the course of the immunization schedule by assessing the area under the curves (AUC) for each animal (Fig. 4). Binding antibody responses towards the BG505 antigen were similar between the three groups (medians between: 53–57) (Fig. 4A). Group 1B had higher binding responses towards the CAP255 trimer (the same antigen this group was vaccinated with; median: 44; interquartile range (IQR): 43–57) as compared to Group 1A (median: 21; IQR: 21–26; p-value: 0.0102) (Fig. 4B). Group 1C had higher binding antibody levels against their vaccine-matched CAP256.SU antigen (median: 45; IQR: 35–52) as compared to Group 1A (median: 25; IQR: 20–30; p-value: 0.0147) (Fig. 4C). All groups had similar binding values to the heterologous 1086C antigen (medians between 31 and 39) with no significant differences. (Fig. 4D). Altogether, immunization of vervet monkeys with diverse SOSIP.664 trimers resulted in a high magnitude of HIV Env-specific binding responses, with some preferential immunogen-matched activity for the groups immunized with subtype C trimers.

### Antibody-dependent cellular phagocytosis activity is elicited after immunization

**Figure 5:**
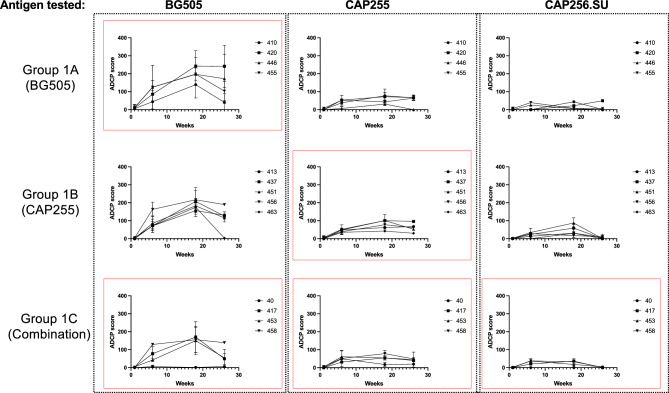
Immunization with SOSIP.664 trimers triggers antibodies with preserved antibody dependent cellular phagocytosis over time. ADCP activity was assessed in plasma from BG505 (Group 1A), CAP255 (Group 1B) and or BG505, CAP255 and CAP456.SU combination (Group 1C) immunized vervet monkeys against homologous (red boxes) and heterologous antigens. ADCP was measured longitudinally before and after the various immunizations. An ADCP score was calculated as the percentage of THP-1 cells that engulf trimer-coated beads multiplied by the geometric mean fluorescence intensity (MFI).

We next examined the ability of the vervet monkey model to elicit Fc effector functions as Fc function has been found to be associated with protection following vaccination in rhesus macaques^24^. We investigated whether antibody dependent cellular phagocytosis (ADCP) was elicited upon vervet monkey immunization with the different SOSIP.644 trimers tested (Fig. 5).

**Figure 6:**
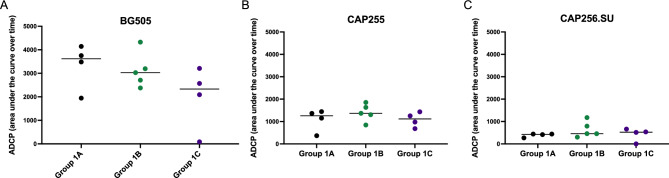
Comparison of antibody dependent cellular phagocytosis responses between immunization groups. ADCP activity was measured and the area under the curve was calculated for each animal across all timepoints and this was compared between Groups 1A, 1B and 1C for each antigen tested: (**A**) BG505, (**B**) CAP255 and (**C**) CAP256.SU. Statistical significance was calculated Graphpad Prism v9.5.1 using the Kruskal–Wallis test with Dunn’s multiple comparison test where *p < 0.05, **< 0.01, ***< 0.001, ****< 0.0001.

We examined the longitudinal ADCP activity against the immunogen-matched SOSIP.664 trimers and found that all the vervet monkeys developed antibodies with ADCP activity (Fig. 5). Group 1C trended towards lower levels of ADCP compared to the others groups throughout the immunization schedule with the lowest responses being towards the CAP256.SU Env protein (Fig. 5). We compared the AUCs for each animal and found that ADCP was similar across the three antigens tested, BG505 (medians between: 2331 and 3617) (Fig. 6A), CAP255 (medians between: 1116 and 1367) (Fig. 6B) and CAP256.SU (medians between: 428 and 525) (Fig. 6C), for all the three groups with no significant differences observed (Fig. 6).

### Vervet monkey Env trimer immunization elicits Tier 1 neutralizing antibody responses

**Figure 7:**
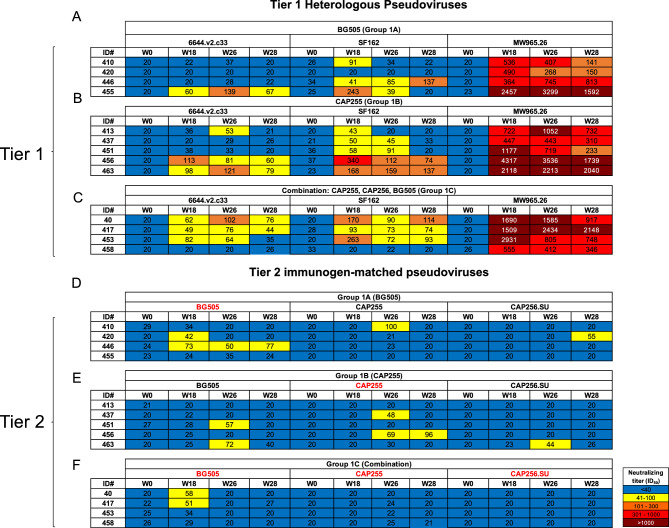
Neutralizing antibody titers following vervet monkey trimer immunizations. Plasma from the pre-immunization bleed and from after the 3^rd^ immunization were tested in our HIV pseudovirus assay for neutralization activity against 6 pseudoviruses; three tier 1 viruses: 6644.v2.c33, MW965.26 and SF162 and three Tier 2 viruses: BG505 + N332 (BG505), CAP255.2000.4D (CAP255) and CAP256.SU. The names in red indicate the vaccine-matched pseudovirus for each vervet monkey group. ID_50_ values are shown with resistance being > 20 (blue) and increasing sensitivity from 20–100 (yellow); 101–300 (orange); 301–1000 (red) and > 1000 (maroon).

Finally, we assessed the neutralizing antibodies in the immunized vervet monkey plasma using a HIV pseudovirus neutralization assay. We tested plasma from baseline, 2 weeks after the third and fourth immunizations and 1 month after the final immunization against Tier 1 pseudoviruses (SF162, 6644.v2.c33 and MW965.26) and the vaccine-matched HIV strains (BG505, CAP255 and CAP256.SU) (Fig. 7). There was no detectable neutralizing activity observed at baseline for any of the animals tested.

One of 4 animals in Group 1A developed neutralizing antibody responses towards 6644.v2.c33 and 3 of 4 animals had SF162-directed neutralizing antibodies. The same trend was observed in Group 1B and 1C where 3 out of the 4 animals in both groups developed responses towards 6644.v2.c33 (Fig. 7A–C). These responses were relatively weak with ID_50_ values between 53–121 for Group 1B (Fig. 7B) and values of 44–102 for Group 1C (Fig. 7C). Both groups had SF162 responses with the highest ID_50_ of 340 being reached after the 3rd immunization in one animal in Group 1B (Fig. 7B). MW965.26 was potently neutralized by the plasma from the all vervet monkeys in Group 1A with ID_50_ values up to 3299 (Fig. 7A). As with Group 1A, both Group 1B and 1C animals had potent antibodies targeting the MW965.26 virus with ID_50_ values up to 4317 for Group 1B (Fig. 7B) and up to 2931 for Group 1C (Fig. 7C).

Only 2 of 4 Group 1A monkeys developed weak, but detectable, autologous (vaccine-matched; indicated in red) neutralizing antibody responses after immunization, with an ID_50_ value of 77 being the highest titer reached (Fig. 7D). Similarly, 2 of 5 Group 1B animals had weak autologous neutralization of the vaccine-matched CAP255 pseudovirus (Fig. 7E). There was sporadic neutralization of the Tier 2, non-vaccine matched pseudoviruses for both Group 1A and Group 1B (Fig. 7D,E). Two Group 1C animals developed weak neutralization towards the BG505 pseudovirus after the third immunization but no other detectable antibody responses were observed in this group for their vaccine-matched pseudoviruses (Fig. 7F).

Altogether, these data indicate that HIV-specific neutralizing antibody responses are elicited by the SOSIP.664 trimer immunization in vervet monkeys, however, they are predominantly targeted at Tier 1 viruses even after four immunizations.

## Discussion

Vervet monkeys have been used extensively to study pathogenesis of various infectious diseases that affect humans including HIV, Ebola virus and SARS-CoV-2^25–27^. In this study, we assessed the feasibility of using vervet monkeys as an animal model to test HIV trimer immunogenicity for pre-clinical testing. We designed and tested a novel subtype C trimer, derived from a PLWH who developed potent, V3/glycan-targeting bNAbs along with the BG505 SOSIP.664 trimer^3^ and the subtype C, CAP256.SU trimer^23^ based on the superinfecting virus which drove the development of potent V2-apex-directed bNAbs in a PLWH^17,18^. We found that the SOSIP.664 immunogens tested in this study elicited robust and cross-reactive binding responses and ADCP activity, however the neutralizing antibody responses were limited to neutralization-sensitive Tier 1 viruses and weak Tier 2 responses.

The vervet monkeys in our study had detectable binding antibodies after just two immunizations which persisted throughout the study period, peaking after the third immunization. The NHPs immunized with either a subtype C trimer alone (Group 1B) or two subtype C trimers in combination with the subtype A BG505 antigen developed more cross-reactive binding responses as opposed to the monkeys who only received the BG505 trimer (Group 1A). These data suggest that subtype C trimers may elicit robust cross-reactive binding responses. However, although high binding responses were observed, the HIV Env trimer contains numerous binding epitopes which are targeted by non-neutralizing antibodies, such as glycan holes and the flexible V3 loop^7,28^. Soluble trimers also have an exposed base region and a large proportion of non-neutralizing responses elicited after trimer immunizations target these areas and are likely the majority of the responses elicited in the vervet monkeys as seen in other primate model studies^9,11^. Mapping these responses using microscopy-based polyclonal epitope mapping techniques would aid in understanding the targets of the binding antibody responses elicited in this animal model^10,29^. In addition, revising the immunization strategy by inhibiting the elicitation of base responses by methods such as blocking the site with an anti-base monoclonal antibody or assembling the trimers on nanoparticles may redirect the immune response towards favorable bNAb targets^30,31^. In addition, priming with a fusion peptide (FP) immunogen followed by trimer boosts has been shown to result in decreased anti-base responses^32–34^. This could be a feasible approach to increase the quality of the neutralizing antibody response in the vervet monkey model.

The presence of ADCP activity suggests the preservation of Fc effector function epitopes in the trimers tested. These responses should be mapped to better understand the ADCP response in this model. However, even if the Fc response is directed towards areas such as glycan holes or the base of the trimer, the antibodies will still be able to perform the recruitment of cytotoxic agents. Although the contribution of ADCP in decreasing the risk of HIV infection in vervet monkeys still needs to be determined, ADCP has been shown to be associated with protection in other NHP models^24,35^. We only tested the ability of the elicited antibodies to perform ADCP but there are other Fc effector functions which are elicited after vaccination such as antibody dependent cellular cytotoxity and complement deposition^36^. Testing these multiple functions in vervet monkey vaccinated plasma may further our understanding of the use of this model to study Fc effector function after vaccination.

The vervet monkey model elicited limited neutralizing antibody responses. The BG505 SOSIP.664 trimer has previously been shown to elicit autologous neutralizing antibody responses in rabbits and macaques^19^. In contrast, we observed only limited autologous neutralizing activity in the vervet monkeys immunized with BG505 alone and even less with BG505 in combination with subtype C trimers. Heterologous neutralization was elicited for all the vervet monkeys tested, regardless of immunogen, but this was targeted towards easy-to-neutralize Tier 1 viruses and likely targeting their exposed V3 loops and other regions which are more open in Tier 1 viruses^37^. Tier 1 viruses are not a good representation of the viruses that are currently circulating in the population which tend to have more resistant neutralization phenotypes^38,39^ and therefore potent responses against Tier 1 viruses are likely not clinically relevant. Despite the findings that the vervet monkey model may not consistently elicit autologous neutralization activity, these data may be affected by factors including the immunogen dosage, number and interval of vaccinations, timing of blood draws and the adjuvant used. As an example, studies which used BG505 DS-SOSIP.664, a version of the BG505 trimer which is more stable than the original SOSIP.664 design, elicited only weak, infrequent autologous responses in macaques and in humans when the adjuvant used was alum^40,41^. This adjuvant is known to be less potent than ISCOMATRIX which is widely used in the HIV immunization field and elicits stronger antibody responses^8,19^. In this study, we used Alhydrogel, an aluminium-based adjuvant. Therefore, further studies using different adjuvants and optimizing other factors may yield increased autologous and Tier 2 heterologous responses.

Subtype C trimers have been shown to be less amenable to the SOSIP.664 design^42^ and therefore the novel CAP255 SOSIP.664 protein, which produced stable trimers, contributes to the small but increasing array of stable, subtype C SOSIP trimers which can be used in immunization studies. The PLWH who the CAP255 virus was isolated from developed a broadly neutralizing antibody response targeted towards the V3/glycan, N332-supersite^16^. This epitope is a favorable vaccine target as neutralizing antibody responses toward it are commonly elicited upon HIV infection^43,44^. The V3/glycan site is now a focus of numerous immunogenicity studies including germline-targeting immunogen research^45–48^. Work is underway in our laboratory to modify the CAP255 SOSIP.664 trimer used in this study to generate a next-generation V3/glycan germline targeting immunogen to be used in prime-boost immunogenicity studies.

There is still a need to identify cheaper and more accessible animal models for pre-clinical testing of HIV vaccine candidates. These models could be implemented in low-resourced countries and would aid in more rapid and widespread testing of immunogens. As cross-reactive binding and Fc effector functioning antibodies were elicited in all the vervet monkeys tested, this model may be adequate for testing antibody functionalities beyond neutralization. However, the limited Tier 2 autologous and heterologous neutralization activity observed in this study suggests further testing of this model will be necessary to determine the utlitity of this model in HIV immunogenicity testing.

## Materials and methods

### CAP255 SOSIP.664 envelope trimer design and expression

The CAP255 envelope sequence was identified and cloned in a separate, unpublished project prior to this study from a PLWH enrolled in the CAPRISA 002 Acute Infection Cohort. We previously performed singe genome amplification on this participant at different timepoints after HIV infection and the CAP255 sequence used in this study was obtained from an early 8 week virus. This study and the previous study to obtain the viral sequence were both approved by the University of the Witwatersrand Human Research Ethics Committee (Medical) under protocol reference number: M210892**.** Participants and their samples from the CAPRISA 002 Acute Infection Cohort were not directly involved in this present study. In addition, the CAP256 trimer that was used was based on a published trimer sequence^23^.

The sequence of a virus isolated 8 weeks after infection (CAP255.2000.4D) was modified into a SOSIP.664 construct as previously described^3^. The codon-optimized construct was ordered from Genscript (Piscataway, NJ, USA), and cloned into a pVRC8400 plasmid. In addition to untagged trimers, we generated C-terminal His-tagged trimers. The HIV trimeric plasmid was co-transfected with furin plasmids in human embryonic kidney (HEK) 293F cells (ThermoFisher Scientific). The supernatant was harvested six days post transfection and purified by affinity chromatography using an agarose-bound *Glanathus nivalus* lectin column for the untagged trimers and a nickel coated sepharose column for the his-tagged trimers. The affinity-purified proteins were further resolved by size exclusion chromatography using the HiLoad 16/600 Superdex 200 pg column at a flow rate of 1 min per ml. The trimer eluted out at approximately 55–65 min. Relevant fractions were passed over a CNBr activated sepharose column coupled with the human monoclonal antibody (mAb) 447-52D, to negatively select the misfolded trimer. The flow-through containing the correctly folded trimer protein was collected, filter-sterilized in a sterile biosafety cabinet and stored at − 80 °C until further use.

### Differential scanning calorimetry (DSC)

Thermal denaturation was studied using a TA (Texas Instruments) Nano DSC calorimeter. The SOSIP.664 trimer concentration was subsequently adjusted to approximately 1 mg/ml. After loading the protein sample into the cell, thermal denaturation was probed at a scan rate of 1 °C/min. Buffer correction, normalization and baseline subtraction procedures were applied before the data were analysed using the manufacturer's NanoAnalyze software. The data were fitted using a 2-state model.

### Animal immunization and blood sampling

Thirteen SIV seronegative adult monkeys matched for age and gender were randomly allocated to three groups (n = 13). Each monkey received immunizations at weeks 0, 4, 16 and 24 with 100 μg total trimer divided into two subcutaneous injections bilaterally on the neck (50 μg of trimer per injection site), either with CAP255 SOSIP.664 (n = 5), BG505 SOSIP.664 (n = 4) or triple combination of trimers (CAP255 SOSIP.664, BG505 SOSIP.664 and CAP256.wk34.c80 SOSIP.RnS2) (n = 4). Immunogens did not contain purification tags. The immunogen was adjuvanted with an equal volume ratio of 2% alhydrogel. Venous blood was obtained at pre-immunization and two weeks after each immunization. A final blood draw was obtained 4 weeks after the final immunization. For immunizations and blood draws, animals were chemically restrained with ketamine hydrochloride at 10 mg/kg bodyweight, which was administered by intramuscular injection. The animal protocol was reviewed and approved by the Ethics Committee for Research Animals of SAMRC (ECRA Ref no.: 09/19). All the methods relating to the animal work were performed in accordance with the relevant guidelines and regulations of this Ethics Committee. In addition, the study is reported in accordance with ARRIVE guidelines.

### Enzyme-linked immunosorbent assay

96-well nickel-coated plates were coated with 2 µg/ml of either CAP255, BG505, CAP256 or 1086C His-tagged trimer and incubated at room temperature for at least 1 h. After washing with 1 × PBS and blocking with a buffer containing 1 × PBS and 5% milk powder, plasma was added at a dilution of 1:100. For monoclonal antibody ELISAs with the CAP255 trimer, antibodies were added at a starting concentration of 10 μg/ml and threefold serial dilutions were conducted. These were incubated for 1 h at room temperature followed by the addition of a horseradish peroxidase-conjugated secondary antibody diluted at 1:3000 and this was incubated for a further 1 h. 3,3′,5,5′-tetramethylbenzidine (TMB) substrate was added and after 5 min the reaction was stopped using 1 M sulfuric acid. Binding was measured at an optical density of 450 nm using a SpectraMax® ABS Plus microplate reader and analysis was conducted in Graphpad Prism v9.3.0.

### HIV pseudovirus-based neutralization assay

HIV-1 pseudotyped viruses were prepared by co-transfection of 293 T cells with *env*-expressing plasmid and the pSG3∆Env backbone plasmid. After a 48 h incubation at 37 °C, 5% CO_2_ supernatant containing pseudotyped viruses was harvested and subsequently stored at − 80 °C until use. Neutralization assays were performed as previously described (Montefiori, 2005). Briefly, pseudotyped viruses were incubated with diluted, heat inactivated plasma samples for 1 h at 37 °C, 5% CO_2_. Thereafter, 1 × 10^6^ TZM-bl cells per ml together with 25 μg/ml of DEAE-dextran were added to each well. The plates were then further incubated for 48 h at 37 °C, 5% CO_2_. Luciferase assay substrate was added to the wells and luminescence was measured. ID_50_ values were calculated as reciprocal dilution of plasma that showed 50% reduction in luminescence compared to the untreated virus control and analysis was conducted in Graphpad Prism v9.5.1.

### Antibody-dependent cellular phagocytosis (ADCP) assay

The BirA-RT biotin protein ligase reaction kit (Avidity) was used to biotinylate Avi-tagged BG505, CAP255 or CAP256 SOSIP trimers as per the manufacturer’s instructions and the ADCP assay was conducted as previously described^35^. Briefly, the biotinylated trimers were coated onto 10 μl fluorescent neutravidin beads. The trimer coated beads were then incubated for 2 h at 37 °C, 5% CO_2_ with a single 1:100 dilution of plasma and titrated five-fold. The beads were further incubated with monocytic THP-1 cell line overnight, fixed with Paraformaldehyde (PFA) (Sigma) and the engulfed beads were detected on the FACSAria II (BD Biosciences). Phagocytic scores were calculated as a percentage of THP-1 cells that engulfed the fluorescent beads multiplied by the geometric mean fluorescence intensity. A no plasma negative control was subtracted to remove background signal. Pooled IgG from HIV-positive donors (HIVIG) (NIH AIDS Reagent programme) and monoclonal antibody CAP256.25 were included as positive controls. Palivizumab was used as a negative control.

### Supplementary Information

Supplementary Figure 1.

## Data Availability

The data from this study are available from the corresponding author on reasonable request.
