# Supplementary material for: Evaluating the antibody response elicited by diverse HIV envelope immunogens in the African green monkey (Vervet) model
Source: Sci Rep. 2024 Jun 10;14:13311. doi: 10.1038/s41598-024-63703-7 (PMC11164991; doi:10.1038/s41598-024-63703-7)
Supplement: Supplementary file 1 — Supplementary Figure 1. [file 41598_2024_63703_MOESM1_ESM.pdf]

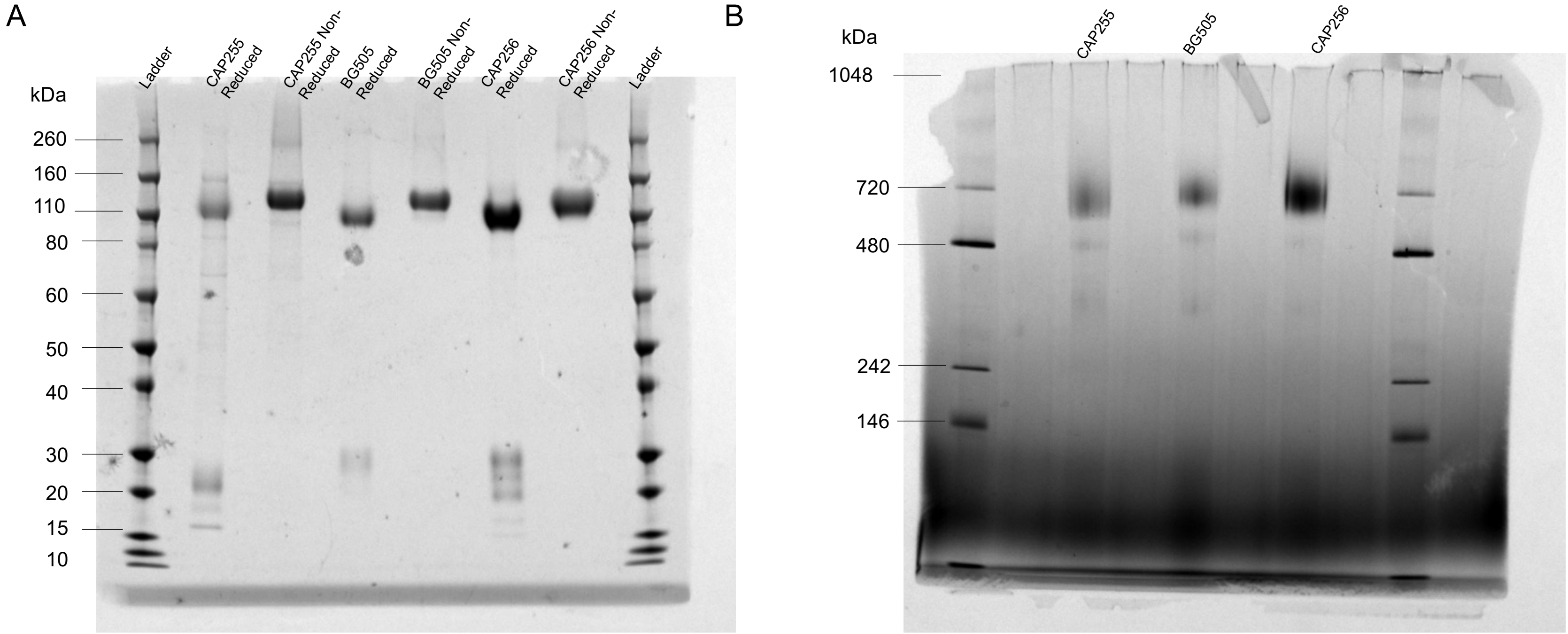

Supplementary Figure 1: Characterization of SOSIP.664 trimers using gel electrophoresis. (**A**) An SDS-PAGE was used to determine the purity of the SOSIP.664 trimers used in this study. Trimers were exposed to both non-reducing and reducing (including beta-mercaptoethanol) conditions. The first and last lanes of the gel contain a ladder to determine the size of the bands observed. (**B**) Native-PAGE was performed on all three SOSIP.664 trimers which were included in the study to determine their size. The first and last lanes of the gel contain a ladder to determine the size of the bands observed.
